# Observations on Medical Education

**Published:** 1813-04

**Authors:** J. W. Empiricis Inimicus

**Affiliations:** Calne


### *To the Editors of the Medical and Physical Journal.*

GENTLEMEN,

THE reading of Senex's Observations on Medical Education, with Censorinus's Letter to Sir Francis Milman, has induced me to state a few sentiments on those points to which each has referred. Although, with Senex, I might be led to ascribe the annual supply of surgeons for our army and navy, demanded by government, as one cause of superficial education, under the present system of unlimited practice in the healing art; yet, that men engaged in the practice of midwifery, should operate as another cause, I cannot reasonably admit. Ought, or ought not, persons following this branch to be well educated, and informed to a certain extent in anatomical knowledge? and, though its practice is, for the most part, uniformly easy, are there not many occasions which demand the utmost fortitude, and most prompt measures? Is this department then meant to be consigned to female hands! Certainly not, because none are better calculated than men well acquainted with the anatomy, physiology, and mutual connections, of parts, than men who are accustomed to meet urgent and dangerous cases in other branches of their profession, and more prepared to meet them.

I coincide with Senex in thinking that there are too many indifferent surgeons, yet attribute not the evil to the same source, but (as was observed by one of our first modern surgeons) to a deficient liberal education, prior to apprenticeship; coming to London with merely a pestle-and-mortar

 knowledge; knowledge; and there not sufficiently impressed with the importance of their profession, and individual interest. Entering the profession, then, with a good education, a full conviction of its importance, with assiduity and perseverance whilst apprentices and hospital students, would do more, I am persuaded, towards eradicating the complained-of evil, than relinquishing midwifery into the hands of women.

Not approving of general asper‹s›ions against any class of men, I consider Senex too severe in supposing practitioners to receive apprentices purely for the sake of premium; for such a supposition attaches to it dishonor, with which, I hope, we are not more tainted than others. Instances, no doubt, might be adduced, substantiating his opinion; and to such I should say, with him, that it would prove no hard task once a week to deliver some practical lesson.

Without a comment on paying bills for stuff, regular quackery, &c. I shall proceed to Censorinus's more interesting letter, which has entered upon a more extensive field of argument, and where much matter might be brought forward on both sides. He who practises surgery alone, seems to be most free from the contention now existing in the medical world. The physician and apothecary, who ought to cultivate a friendly union, are now most at variance. One complaining of infringements and attempts at usurpation, the other of great privations. Which party is culpable, cannot easily be determined, as their grievances appear coeval, perhaps coequal. Rather than argue on this the least important part of the subject, I would argue for a reconciliation, and should be happy could such be effected. Taking a general survey, you will find the injured physician endeavoring to diminish and weaken the apothecary, by ordering his prescriptions to a chemist and druggist; you will find the injured apothecary rallying to seek redress from parliament; whilst the chemist and druggist, relishing the dispensing of medicines, will retain it by every means in his power.

That each branch ought to perform their duties without any attempts at encroachment, I hope, will be admitted as reasonable; and, admitting it, I wish to mention their several callings, to propose one question, and conclude with measures for a general good understanding. The physician, a man entitled to our highest respect, is called upon in urgent cases, to tender his best advice, write a prescription, and receive his fee. An apothecary (supposing him what he ought to be—well educated and regularly bred), visits patients before, with, and after, the physician, and has, or ought to have, his prescription to dispense. The chemist and and druggist ought only to prepare the various articles used, and vend them. If apothecaries were not to visit and prescribe, who would be found attending that immense class of people which cannot afford to render the physician his fee? That is the question I would ask. But the apothecary complains of inadequate remuneration, and, as Censorinus expresses it, is endeavoring to legalize the demand of fees. That some remuneration for trouble ought to be granted, is as reasonable, as regularly to demand fees is unreasonable. In order, therefore, to adjust disputes, and to rescue the profession from that degradation and ignorance into which it is supposed to have fallen, I conceive the adoption of some substantial and enforcing measures necessary.

I should propose, that, whatever branch of the profession a youth was inclined to pursue, he should produce testimonials of his scholastic acquirements, also testimonials from his master after serving an apprenticeship, and again his hospital certificates prior to an examination. Secondly, that every person found practising medicine, surgery, &c. without producing the above-mentioned credentials of his abilities and power, might be reported to those tribunals—the Colleges of Physicians and Surgeons, and that these should be vested with full power to prevent such persons from practising. And thirdly, that a re-union might be effected by proper assurances being given to physicians, that no wish exists of entrenching upon their privileges, or assuming their character; and, on the other hand, by physicians directing their prescriptions into their former proper channels. Under such a system, we should not be so oppressed by numbers, the public not so deluded by imposters; but that harmony, prosperity, and advancement of science, would be its happy consequences.

I remain, Gentlemen,

*Calne,* Your most humble Servant,

